# Contrast-Enhanced Ultrasound in the Diagnosis of Solid Renal Lesions

**DOI:** 10.3390/jcm13133821

**Published:** 2024-06-29

**Authors:** Monika Zbroja, Maryla Kuczyńska, Katarzyna Drelich, Eryk Mikos, Agata Zarajczyk, Mateusz Cheda, Izabela Dąbrowska, Anna Drelich-Zbroja

**Affiliations:** 1 Department of Pediatric Radiology, Medical University of Lublin, 20-090 Lublin, Poland; 2 Department of Interventional Radiology and Neuroradiology, Medical University of Lublin, 20-090 Lublin, Poland; 3 Students’ Scientific Society at the Department of Pediatric Radiology, Medical University of Lublin, 20-090 Lublin, Poland; 4 Students’ Scientific Society at the Department of Interventional Radiology and Neuroradiology, Medical University of Lublin, 20-090 Lublin, Poland

**Keywords:** CEUS, renal lesions, diagnostic imaging

## Abstract

The availability of imaging methods has enabled increased detection of kidney lesions, which are a common clinical problem. It is estimated that more than half of patients over the age of 50 have at least one undetermined mass in the kidney. The appropriate characterization and diagnosis of lesions imaged in the kidney allows for proper therapeutic management. Previously, contrast-enhanced computed tomography (CT) and contrast-enhanced magnetic resonance imaging (MRI) have been used in their extended diagnosis. However, the limitations of these techniques, such as radiation exposure, renal toxicity, and allergies to contrast agents, must be considered. Contrast-enhanced ultrasound (CEUS) is increasingly being used as an examination to resolve interpretive doubts that arise with other diagnostic methods. Indeed, it can be considered both as a problem-solving technique for diagnosing and distinguishing lesions and as a technique used for observation in preservative treatment. Evaluation of the enhancement curve over time on CEUS examination can help to differentiate malignant renal cell carcinoma (RCC) subtypes that should be resected from benign lesions, such as oncocytoma or angiomyolipoma (AML), in which surgery can be avoided. It allows for distinguishing between benign and malignant tumors, renal and pseudotumors, and solid and cystic tumors. Therefore, with recent advances in ultrasound technology, CEUS has emerged as a fast, reliable, and cost-effective imaging tool in the preoperative evaluation and diagnosis of solid renal masses.

## 1. Introduction

The availability of imaging methods has enabled increased detection of kidney lesions, which are a common clinical problem [1]. It is estimated that more than half of patients over the age of 50 have at least one undetermined mass in the kidney [2,3].

Solid renal masses are characterized by having little or no fluid. They consist mainly of vascularized tissue (i.e., elements that are enhanced after administration of exogenous contrast agents) [4]. Despite their lower incidence compared to cystic lesions, up to 90% of solid masses are malignant [5]. The risk of malignancy correlates with size; it usually appears in about 50% of lesions smaller than 1 cm and more than 90% of masses larger than or equal to 7 cm [5].

The most diagnosed renal malignant masses in clinical practice are RCC, urothelial carcinoma, lymphoma, and metastases, while the most common benign renal masses are AML, oncocytoma, and inflammatory pseudotumors or pseudotumors [5].

The appropriate characterization and diagnosis of lesions imaged in the kidney allows for proper therapeutic management. Previously, contrast-enhanced CT and contrast-enhanced MRI have been used in their extended diagnosis [6]. However, the limitations of these techniques, such as radiation exposure, renal toxicity, and allergies to contrast agents, must be considered [7]. Therefore, with recent advances in ultrasound (US) technology, CEUS has emerged as a fast, reliable, and cost-effective imaging tool for the preoperative evaluation and diagnosis of solid renal masses [7].

The method used to manage solid renal tumors is ablation, which is a percutaneous procedure that destroys tumor tissue. CEUS can also be used to evaluate the effectiveness of this treatment method.

The purpose of this review is to provide an updated literature overview of the potential use of CEUS in the diagnosis of solid renal masses.

## 2. Methodology

The main data sources used to carry out the literature search were three bibliographic databases: PubMed, Medline, and Embase. The literature search was limited to the English language by combining the topic-related keywords “renal”, “solid”, “mass”, “CEUS”, and “microflow”, using Boolean operators. The electronic databases were electronically searched from April 2018. The reference lists of relevant articles were hand-searched for additional relevant studies.

Studies published within the last 6 years (as of 2018) were included, as it was recognized that significant advances in ultrasound techniques and improvements in diagnosis and definition in imaging renal lesions using CEUS made these older studies less relevant. In addition, both EFSUMB and WFUMB have not published updates to their guidelines regarding non-hepatic applications of CEUS. Unpublished studies, non-peer-reviewed studies, conference abstracts, letters to the editor, and opinion articles were excluded. If data from a single study population was reported more than once, the publication with the most complete information was included. After the review process, 60 references were obtained.

## 3. Application of CEUS

CEUS is increasingly being used as an examination to resolve interpretive doubts that arise with other diagnostic methods. Indeed, it can be considered both as a problem-solving technique for diagnosing and distinguishing lesions and as a technique used for observation in preservative treatment [8].

The development of ultrasound contrast agents (UCAs) improved the visualization of complex vascular structures, overcoming some of the limitations of B-mode and Doppler imaging [9]. Among others, we can distinguish Optison, Definity/Luminity, Sonazoid and SonoVue; however, the last one is mostly used [9]. UCAs are not nephrotoxic and cannot be excreted by the kidney; therefore, they can be safely administered to patients with renal failure without the risk of contrast-related nephropathy or nephrogenic systemic fibrosis [10,11]. Multiple injections of contrast agent are permitted, due to its short half-life (about 5 min) [12]. CEUS shows real-time blood flow using microbubble contrast agents and complementary harmonic pulse sequences [11].

Compared to contrast-enhanced CT, CEUS is more sensitive due to its strong inhibition of background tissue. This makes it possible to distinguish solid oligovascular masses from atypical cystic masses and easier to visualize septa and enhancing solid elements [2,13]. CEUS can identify more septa, provide a better assessment of their thickness, and more frequently detect solid components within cystic lesions [14].

CEUS’s limitation is the relatively reduced number of frames per second of angiographic images, even though it is considered as a dynamic method. In the case of a small, richly vascularized tumor, the perfusion process in the arterial phase of CEUS is very fast. Consequently, it is difficult to accurately identify the tumor perfusion process and blood vessel morphology, which limits diagnostic effectiveness [2].

CEUS examination may be able to depict the perfusion process more clearly and accurately using high frame frequency contrast-enhanced ultrasound imaging technology. Compared to current CEUS, which uses a frequency of 10–15 Hz, the frequency of H-CEUS is increased to 50–80 Hz. This allows the adverse effects of low frame rate on the temporal resolution of contrast images to be ameliorated to some extent, thereby providing more diagnostic information [2,13]. When it comes to the kidney, CEUS focuses on the renal cortex and medulla within the region of interest (ROI), obtaining renal microcirculatory perfusion parameters from a perfusion curve model based on intensity-over-time data [15].

## 4. Solid Renal Lesions in CEUS

Solid renal lesions can be malignant or benign. Statistics indicate that solid masses are a significant minority in the number of all diagnosed renal lesions, but they comprise 90% of solid renal tumors that are malignant [16]. Statistically, about 70% of renal malignancies are RCC [2]. It is the most lethal malignant neoplasm of the genitourinary system, which originates in the tubular epithelium of the renal parenchyma [17]. Among RCC diagnoses, clear cell carcinoma accounts for the majority, while papillary and chromophobe renal cell carcinoma occupy 10–15% and 5% of all RCC diagnoses, respectively [18]. The most common benign solid tumors of the kidney are AML and renal oncocytoma [18]. AMLs are hamartomatous benign tumors composed of newly formed vessels, smooth muscle cells, and well-differentiated adipose tissue [19]. About 80% of AMLs occur as isolated entities, but they can also be associated with the tuberous sclerosis complex [20]. Abundant adipose tissue is a characteristic feature that can be visualized by other imaging modalities such as CT and MR. However, it is sometimes too scarce to be detected. This presents a difficulty in distinguishing AML from RCC in CT or MR [20]. Oncocytoma is a benign epithelial neoplasm composed of oncocytes and originating from the cells of the distal renal tubules [21]. Once a malignant lesion is initially diagnosed, in order for appropriate treatment to be undertaken, it is very important to assess the grade and histological classification of RCC.

Contrast-enhanced ultrasonography is most often used as a complementary method to other imaging modalities in the evaluation of renal lesions. This examination is performed using a microbubble contrast agent that is not nephrotoxic. CEUS is performed especially in patients with chronic renal failure or allergy to contrast agents used in other imaging modalities such as CT or MRI [22,23].

Accurate preoperative diagnosis of solid renal masses has a significant impact in assessing the patient’s prognosis and treatment choice. Evaluation of the enhancement curve over time in CEUS examination can help to differentiate malignant RCC subtypes that should be resected from benign lesions, such as oncocytoma or AML, in which surgery can be avoided. We can distinguish between clear cell RCC, papillary RCC, and chromophobe RCC, which are among the most common histological subtypes of RCC [16].

In studies conducted by various researchers, different parameters have been considered in the evaluation of solid lesions during CEUS examination. These include enhancement of the lesion compared to surrounding healthy renal parenchyma, homogeneous or heterogeneous wash-in, and the presence or absence of a pseudo-capsule, defined as rim enhancement [22,24].

The results of studies conducted in the recent past have confirmed that the presence of a pseudo-capsule, rapid wash-out, and high and heterogeneous enhancement on CEUS examination indicate the malignant nature of the lesion and most often indicate a diagnosis of renal cell carcinoma [19,22,24]. In contrast, benign lesions usually show slow centripetal enhancement, homogeneous peak enhancement, and gradual wash-out [19,24].

**Figure 1:**
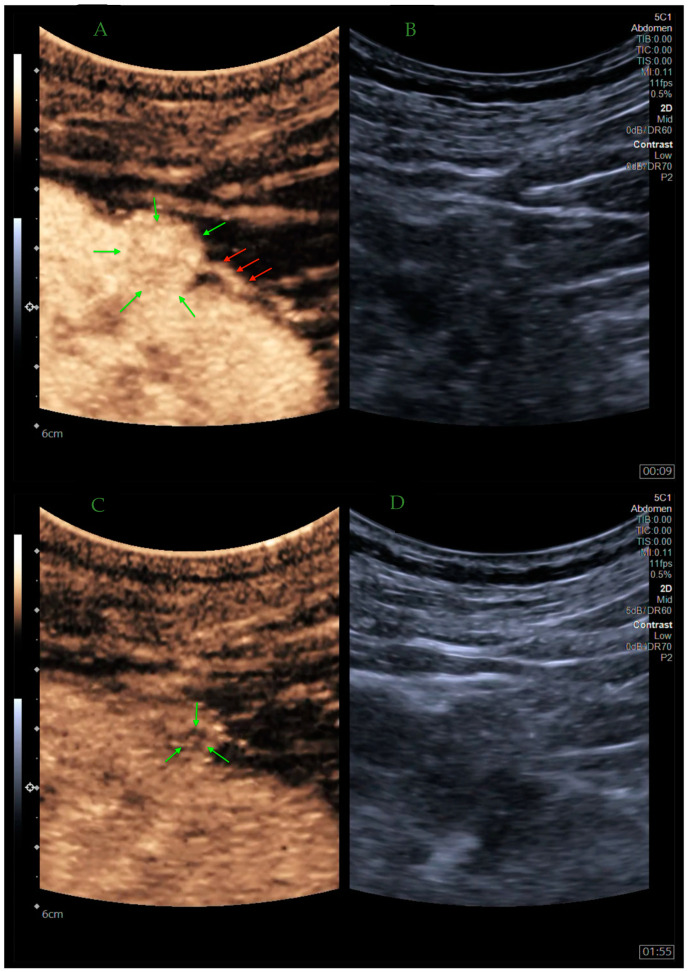
CEUS. Vivid, chaotic hyperenhancement of the lesion (green arrows) is visible in the early arterial phase. Pathologic feeding vessel (red arrows) is visible right next to the lesion further confirmed as an RCC (**A**,**B**). Pathological wash-out is visible in the central part of the lesion, as compared to uniformly enhanced normal renal parenchyma (green arrows) (**C**,**D**) [own source].

Malignant lesions on CEUS examination are most often characterized by heterogeneous enhancement, rapid wash-out, and the presence of a pseudo-capsule sign (Figure 1). By contrast, benign lesions usually show slow centripetal enhancement, homogeneous peak enhancement, and gradual wash-out [19]. Taking into account the different histotypes of both benign and malignant lesions, a typical picture can be presented on CEUS examination.

Clear cell RCC is enhanced earlier and to a greater extent than the normal renal cortex due to a shorter rise time and time to peak, a steeper wash-in slope, and a higher peak intensity than normal renal cortex [25]. According to authors from Memorial Sloan Kettering Cancer Center in New York, CEUS has 63% sensitivity and 80% specificity in distinguishing ccRCC from low-grade malignant and benign tumors [26]. Papillary RCC is enhanced later and to a lesser extent than the normal renal cortex, whereas chromophobe RCC, on the other hand, shows similar enhancing relative to the cortex [27]. In addition, it is hypoenhanced relative to clear cell RCC. Benign lesions such as oncocytoma and AML tend to be enhanced earlier and to a greater extent than the renal cortex [27]. In Gerst et al., examination of two out of three oncocytomas showed early hyperenhancement, whereas Wu et al. described five oncocytomas [26,28]. A comparison between different RCC histotypes and AML is shown in Table 1.

CEUS is a useful assessment tool for the vascularization of lesions. Sun D. et al., in their study comparing four renal tumor histotypes (clear cell carcinoma, “low-grade malignant renal cell cancers”, urothelial carcinoma of the renal pelvis, renal angiomyolipoma) by CEUS examination, unambiguously found that clear cell carcinoma of the kidney is significantly different from the other three histotypes. In particular, clear cell carcinoma shows differentiation in terms of peak intensity, time of wash-in, and peak. This is due to the characteristic rich microvasculature of the lesion, as well as the presence of large and irregular vessels that are distorted [18].

In a study conducted by Tufano A. et al. on CEUS in the evaluation of renal lesions with histopathology validation, the results suggested that this examination is a promising additional diagnostic technique capable of distinguishing malignant and benign renal masses [24].

## 5. Cystic vs. Solid Renal Lesions in CEUS

Renal cysts are mostly detected incidentally when performing abdominal imaging examinations and are more common than solid lesions [30]. They can be divided into simple and complex ones. A simple cyst is observed accidentally around 41% of cases and is defined as a mass with fluid content with a thin (≤2 mm) and well-defined wall with thin-walled anechoic masses in conventional US [30,31]. Complex cysts appear usually secondary to the presence of intracystic hemorrhage or infection [30]. Another division can be made into benign and malignant renal lesions. The first group includes simple cysts, hemorrhagic and proteinaceous cysts, local cystic kidney ailment, multicystic dysplasia, and others, whereas the second type includes RCC and cystic clear cell carcinoma [31]. Nearly 15% of clear cell carcinomas have cystic variation and there are certain histological processes that can characterize cystic renal cell carcinoma, including unilocular and multilocular cystic growth or cystic necrosis that can also be observed as imaging patterns [31].

Large or fast-growing solid renal masses usually show a more aggressive nature. Cystic masses are different due to the fact that the nonenhancing part of the mass might grow; however, it does not change the risk of progression [32]. Nevertheless, they still may cause some serious complications, from non-neoplastic conditions to benign and malignant tumors, which is why proper assessment of imaging features and the nature of the cyst is important in the selection of proceeding options.

The classification of cystic renal lesions, called the Bosniak Classification system, is assigned to reflect the interpretation, with an increasing likelihood of malignancy. Over time, the significance of CEUS has improved in the assessment of cystic renal lesions, especially those with an unclear cystic nature, so a modification of the classification was introduced for use in CEUS examination. Table 2 introduces the Bosniak subdivision for the CEUS version. According to EFSUMB 2020 guidelines, while characterization of simple cysts (category I) and of a subgroup of minimally complicated benign cysts (category II) is obtained on B-mode US, the majority of complex renal cysts are effectively characterized on CEUS [33,34].

Additionally, one study showed that CEUS is superior to CT for imaging malignant renal tumors [35]. In this study, five lesions were correctly diagnosed as Bosniak category III or IV by CEUS but were misdiagnosed by CT and the solid component within three lesions supported diagnostic suspicion of malignant cystic tumor in CEUS not in CT. The conclusion was that the diagnostic accuracies of CT and CEUS for malignant renal tumors were 74% and 90%, respectively [35]. A study by Najafi A. et al. aiming to demonstrate the utility of CEUS to characterize initially unclear kidney lesions in everyday clinical settings showed that, of 301 cystic lesions, 262 were correctly classified as benign and 27 as malignant, with only 12 lesions falsely classified as malignant [30]. CEUS may be useful for further characterization of renal lesions with indeterminate enhancement on CT due to better visualization of tumor microvessels [36]. In addition, a common finding in complex cystic lesions is the detection of microbubbles displaced in the septa; however, this should not be interpreted as a sign of malignancy [30]. According to Elbanna K.Y. et al., CEUS may detect higher Bosniak types compared to CT/MRI, even in 40.4% [37,38].

**Table 2:** Bosniak renal cyst classification based on CEUS [39].

Bosniak Score	CEUS Appearance
I	Thin wall without irregularities with no enhancement on CEUS, or individual microbubbles running within tiny vessels in the wall.
II	Thin wall and septa without irregularities with no enhancement, or individual microbubbles running within tiny vessels in the wall and septa.
IIF	Thin or minimally thickened (2–3 mm), multiple septa without irregularities with no enhancement, smooth or minimally thickened wall.
III	Enhancing smooth thick (≥4 mm) wall or septa, and/or enhancing irregular (>3 mm) walls and/or septa. No nodules are seen.
IV	Enhancing smooth thick (≥4 mm) wall or septa, and/or enhancing irregular (>3 mm) walls and/or septa. Enhancing soft-tissue protrusions, either nodules with obtuse margins (≥4 mm) or with acute margins of any size.

## 6. Differentiation between Renal Tumors and Pseudotumors in CEUS

Renal pseudotumors composed of non-neoplastic tissue pose a diagnostic challenge due to the imitation of tumors at imaging. The main criteria for the diagnosis of pseudotumors is B mode isoechogenicity compared to the surrounding renal cortical parenchyma and renal vessel branching according to the architecture of the renal parenchyma on color Doppler. However, in some cases, additional diagnostic methods are required to distinguish renal pseudotumors from neoplastic tissue.

CEUS offers an immediate alternative to referring patients for CT or MRI, clarifying ambiguous lesions diagnosed by B-mode ultrasound.

Renal pseudotumors with regular vascular architecture are characterized by remaining isoechoic to normal renal parenchyma in all phases of enhancement after ultrasound contrast agent administration [40,41,42]. However renal tumors are known to show different patterns of contrast enhancement with early enhancement in the arterial phase or late wash-out phase [41]. This feature corresponds to about 95% of cases [41].

Acute focal pyelonephritis is also a type of renal pseudotumor. The diagnosis is based on clinical examination and laboratory findings, while CEUS examination is used to confirm the diagnosis. Acute focal pyelonephritis, compared to neoplastic lesions, is characterized by the vascular architecture of the normal parenchyma visible in the early arterial phase, with branching from the hilum to the periphery [43]. The validity of the diagnosis is also confirmed by regression of the lesion during follow-up examinations [44].

## 7. CEUS vs. Other Diagnostic Imaging Methods

**Figure 2:**
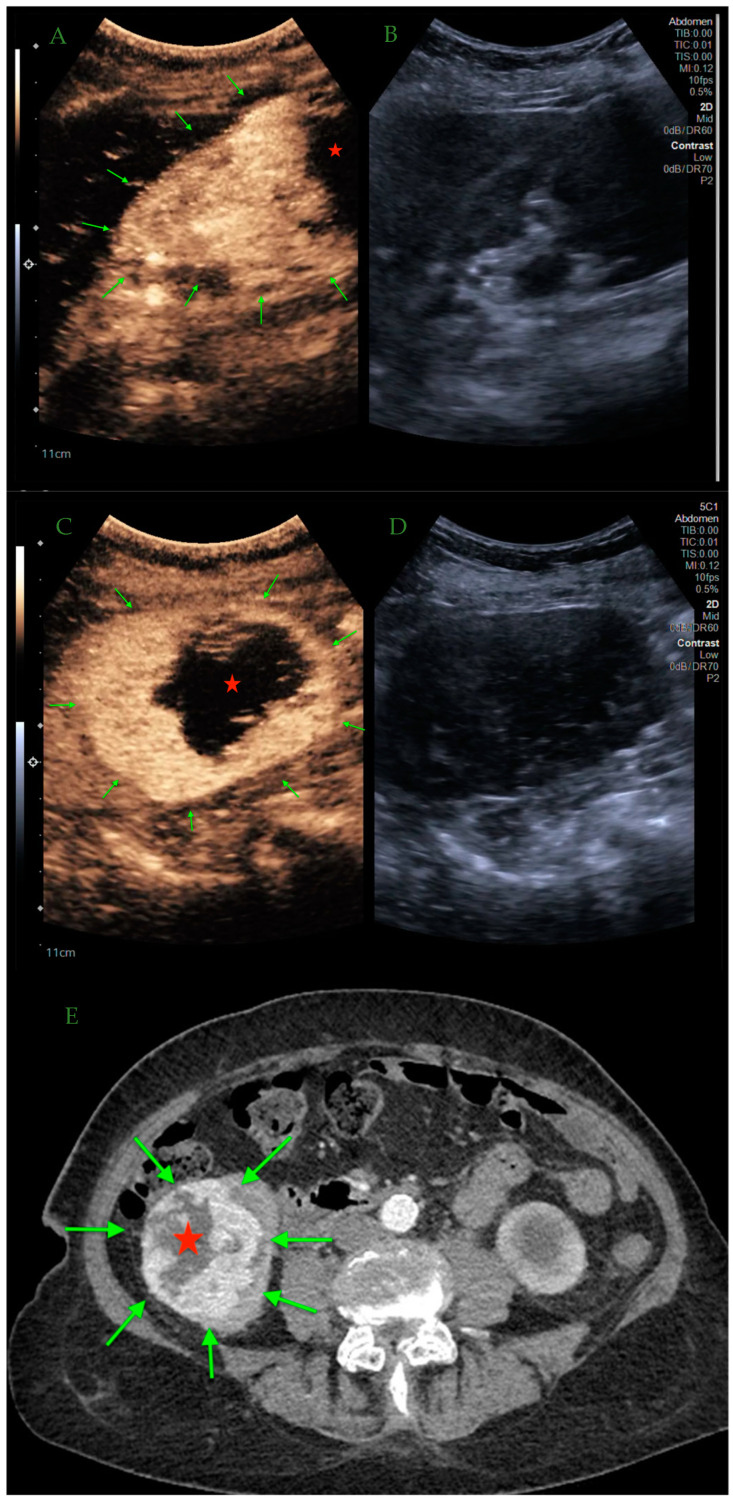
CEUS—RCC. Rapid, disorganized pattern of hyperenhancement of the RCC (green arrows) in the early arterial phase with marginal unenhanced region of necrosis (red star) (**A**,**B**). Clear boundaries of the lesion (green arrows) with a large area of internal necrosis (red star) can be appreciated at least as well as in CE-CT (**C**–**E**) [own source].

**Figure 3:**
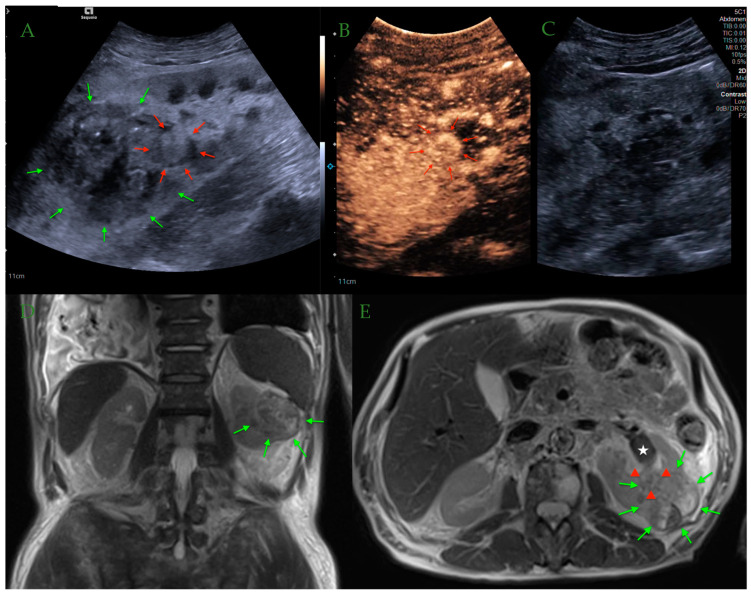
Heterogeneous B-mode appearance of the upper pole right renal lesion (green arrows) suspected of malignancy. Distended intrarenal venous branch (red arrows) with internal echo is visible as well—the differentia includes bland and malignant thrombus (**A**). On CEUS, vivid enhancement of both the renal lesion and intravenous echogenic material (red arrows) can be appreciated, indicating a malignant process infiltrating a vascular (venous) branch (**B**,**C**). T2w coronal magnetic resonance (MR) images showing the heterogenous left renal tumor consistent with RCC (**D**). On axial T2w MR scans, the segmental malignant vein thrombus (red arrowheads) and distended left renal vein (white star) with no obvious infiltration can be appreciated on the background of primary malignancy (red arrows). This is in keeping with previous CEUS findings (**E**) [own source].

The greater availability of imaging examinations has contributed to an increase in the number of incidentally detected lesions in organs, including the kidney. Although CT and MRI are mostly the modalities of choice used for final diagnosis of suspected renal lesions, CEUS is gaining increasing diagnostic importance (Figure 2 and Figure 3). While in CT there is ionizing radiation exposure and in MR there is the need for sedation or general anesthesia, CEUS remains a safer and less invasive option.

According to Mostafa Atri et al., CEUS has the highest contrast resolution of any clinical imaging modality [23]. One injection includes approximately 10^8^ microbubbles and each of them can be visualized, so it is possible to depict microflows within even very small lesions. Additionally, it exhibits strong background tissue inhibition, which makes it more sensitive than enhanced CT in showing very scant blood flow and differentiating between solid oligovascular masses and atypical cystic masses [13]. The difference also concerns contrast agents. Currently available CT and MRI contrast agents diffuse into the interstitial space, which distinguishes them from US contrast agents, which are purely intravascular. Additionally, they introduce a low incidence of side effects, the most severe of which is anaphylactic reaction, which according to WFUMB/EFSUMB recommendations has an incidence rate of 0.014% (considerably lower than that with iodinated CT—0.035–0.095%— or gadolinium-based MRI contrast agents—6.3%) [45,46]. As they do not contain iodine, they do not interact with thyroid function and can be used when renal function is compromised, and they do not lead to contrast-agent nephropathy [47]. An important feature is that CEUS of the kidney can be performed with a lower dose of contrast agent due to higher perfusion and the more superficial location of the kidney [23]. Although CEUS of the kidney is still in the consideration phase for pediatric patients, EFSUMB recognizes that it has potential for comparable pediatric renal indications [48]. In one of the case series mentioned, nine children with renal failure, who could not be given CT or MRI contrast agents, underwent CEUS evaluation and the authors found cystic lesions, renal angiomyolipoma, nephronia, renal abscesses, pseudotumors, and renal cell carcinoma, which were proven histopathologically [48,49].

Four-dimensional MR and 4D CT techniques that produce three-dimensional images of the body’s internal structures with the added dimension of time also have diagnostic significance in planning and monitoring tumors [50,51]. According to one study, 4D CT is a promising tool in diagnostics of RCC [51]. The results revealed significant heterogeneity in RCCs, as shown by both qualitative and quantitative perfusion analyses, primarily due to the presence of necrotic tumor areas. Although 4D-MRI characterizes motion artifacts, it often lacks the contrast to clearly visualize the tumor, unlike CEUS [50].

Additionally, ultrasound flow imaging examination, i.e., microflow imaging (MFI)—Canon modality— or superbmicrovascular imaging (SMI)—Philips modality, is gaining diagnostic significance. MFI can detect the low-speed blood flow and small blood vessels of renal tumors and improve the peripheral annular blood flow display rate of malignant tumors; however, CEUS better displays the internal and peripheral enhancement of masses according to Bertolotto et al. [52,53]. There are a few articles suggesting the usage of CEUS combined with MFI. The study by Mao et al. showed that, in the malignant tumor group, SMI examination of three tumors showed no obvious blood flow signal; however, CEUS-MFI showed fine vessels extending from the periphery to the center [54]. Additionally, CEUS was better in the examination of masses with the largest diameter less than 2 cm, deep location, or dorsal renal masses. Furthermore, another study by Kuang et al. inferred that CEUS-MFI had the highest consistency with pathological diagnosis (Kappa = 0.808) in comparison to MFI alone [55]. Overall, it can be concluded that CEUS can detect more blood flow signals than SMI.

Although CEUS has many advantages, there are also some limitations. CEUS is not efficient in the case of bowel gas and obesity of patients [56]. It is unable to provide a simultaneous view of both kidneys, as is the case in CT and MRI imaging [8]. Moreover only one lesion can be evaluated at a time and repeated bolus administration of UCA is required in order to assess another one [57]. The comparison between CEUS, CT, and MRI is shown in Table 3.

## 8. Monitoring Tumor Ablation

In recent years, percutaneous ablative procedures, such as radiofrequency, microwave ablation, and cryoablation, have been increasingly used as an alternative to either partial or complete nephrectomy for treatment of small renal masses [58]. Ablation probes are usually placed under sonographic guidance; however, sometimes B-mode US is not enough, especially when taking into consideration very small lesions. According to Fang Guo et al., CEUS has been widely used for post-radiofrequency ablation imaging due to its advantages of high performance, excellent safety profile, low cost, and possibility for use in patients with renal failure [59]. Thus, CEUS is the preferrable method to detect ablation sites and guide the probe. Additionally, the time after the procedure is extremely important to assess whether the intervention has succeeded or not. The treatment is successful when the lesion becomes completely avascular. Evaluation of ablative treatment requires monitoring of the ablated area in search of possible complications. CEUS is used for follow-up, especially early after the procedure [8]. According to one meta-analysis, the diagnostic accuracies of CEUS (with CT or MRI as a reference) <6 weeks after ablation were 90.2% for pooled sensitivity and 99.3% for pooled specificity, whereas >6 weeks, they were 95.3% for pooled sensitivity and 97.6% for pooled specificity [60].

## 9. Conclusions

Over the past few years, the importance of CEUS has definitely increased as the preferable modality in the evaluation of solid masses in the kidney. CEUS offers excellent spatial and temporal resolution and allows for real-time dynamic imaging of blood flow, which is crucial in the diagnosis of solid lesions. This allows for accurate assessment of the lesion’s vascularization characteristics, which is of significant diagnostic importance. Additionally, it allows for distinguishing between benign and malignant tumors, renal and pseudotumors, and solid and cystic tumors. This imaging technique offers a compelling alternative to both CT and MR examinations due to its non-ionizing radiation, no requirement for sedation or general anesthesia, and no impact on kidney or thyroid function. Moreover, UCAs introduce a low incidence of side effects when compared to iodinated CT or gadolinium-based MRI contrast agents. Many examinations confirmed that CEUS has the highest contrast resolution. It also reduces the need for CT scans, cutting down on radiation exposure and sparing patients potential renal toxicity concerns. It can distinguish solid oligovascular masses from atypical cystic masses and more easily visualizes septa and enhancing solid elements than CT. When compared to MRI, it also presents a more economical option for our healthcare system, alleviating financial strain. CEUS better displays internal and peripheral enhancement of masses when compared to SMI/MFI. It is a safe, practical, and relatively cheap modality for the evaluation of solid masses in the kidney. Due to the short half-life of its contrast agents, more frequent use is possible. CEUS can be used for regular follow-up, monitoring, and evaluating the effectiveness of treatment methods. However, for now, it is most often used as a complementary method to other imaging modalities in the evaluation of renal lesions.

## Figures and Tables

**Table 1:** Evolution of enhancement features in CEUS for different RCC subtypes and angiomiolipoma (own processing based on the source) (Table 1) [29].

Type of Tumor	Peak Intensity	Mean Transit Time	Tumor to Cortex Intensity
ccRCC	+++	+	+++
pRCC	+	+	+
chRCC	+	+	+
AML	++	++	++

ccRCC—clear cell renal cell carcinoma, pRCC—papillary renal cell carcinoma, chRCC—chromophobe renal cell carcinoma, AML—angiomyolipoma; +++—higher, ++—medium, +—lower.

**Table 3:** CEUS, contrast-enhanced MR, and contrast-enhanced CT comparison.

Modality	CEUS	CE-MR	CE-CT
Radiation exposure	No radiation	No radiation	Radiation exposure
Toxicity	Nontoxic to kidneys	Renal toxicity	Renal toxicity
Duration	Fast time modality	Long time modality	Fast time modality
Contrast half-life	Short half-life	Long half-life	Long half-life
Contrast resolution	Highest contrast resolution	Lower contrast resolution	Lower contrast resolution
Operator dependence	Operator-dependent	Operator-independent	Operator-independent
Expense	Cheaper examination	More expensive examination	More expensive examination

## Data Availability

No new data were created.

## References

[1] Capitanio U., Bensalah K., Bex A., Boorjian S.A., Bray F., Coleman J., Gore J.L., Sun M., Wood C., Russo P. (2019). Epidemiology of Renal Cell Carcinoma. Eur. Urol..

[2] Zhang W., Wang J., Chen L. (2024). Characteristics of high frame frequency contrast-enhanced ultrasound in renal tumors. BMC Med. Imaging.

[3] Barr R.G., Peterson C., Hindi A. (2014). Evaluation of indeterminate renal masses with contrast-enhanced US: A diagnostic performance study. Radiology.

[4] Silverman S.G., Israel G.M., Herts B.R., Richie J.P. (2008). Management of the incidental renal mass. Radiology.

[5] Kay F.U., Pedrosa I. (2018). Imaging of Solid Renal Masses. Urol. Clin. N. Am..

[6] Yoo J., Lee J.M., Kang H.J., Bae J.S., Jeon S.K., Yoon J.H. (2023). Comparison Between Contrast-Enhanced Computed Tomography and Contrast-Enhanced Magnetic Resonance Imaging with Magnetic Resonance Cholangiopancreatography for Resectability Assessment in Extrahepatic Cholangiocarcinoma. Korean J. Radiol..

[7] Tufano A., Antonelli L., Di Pierro G.B., Flammia R.S., Minelli R., Anceschi U., Leonardo C., Franco G., Drudi F.M., Cantisani V. (2022). Diagnostic Performance of Contrast-Enhanced Ultrasound in the Evaluation of Small Renal Masses: A Systematic Review and Meta-Analysis. Diagnostics.

[8] Granata A., Campo I., Lentini P., Pesce F., Gesualdo L., Basile A., Cantisani V., Zeiler M., Bertolotto M. (2021). Role of Contrast-Enhanced Ultrasound (CEUS) in Native Kidney Pathology: Limits and Fields of Action. Diagnostics.

[9] Sridharan A., Eisenbrey J.R., Forsberg F., Lorenz N., Steffgen L., Ntoulia A. (2021). Ultrasound contrast agents: Microbubbles made simple for the pediatric radiologist. Pediatr. Radiol..

[10] King K.G. (2020). Use of Contrast Ultrasound for Renal Mass Evaluation. Radiol. Clin. N. Am..

[11] Dong S., Zhang B., Chen Z., Du D., Shi H., Zhao Y., Tang Y., Luo H., Jiang J. (2024). Assessment of renal quality with quantitative contrast-enhanced ultrasound (CEUS) for differentiating kidney histopathology before procurement. Int. J. Med. Sci..

[12] Giangregorio F., Garolfi M., Mosconi E., Ricevuti L., Debellis M.G., Mendozza M., Esposito C., Vigotti E., Cadei D., Abruzzese D. (2023). High frame-rate contrast enhanced ultrasound (HIFR-CEUS) in the characterization of small hepatic lesions in cirrhotic patients. J. Ultrasound.

[13] Aggarwal A., Goswami S., Das C.J. (2022). Contrast-enhanced ultrasound of the kidneys: Principles and potential applications. Abdom. Radiol..

[14] Chen R., Gao B., Wang X., Zhao H., Wang X., Liu D. (2024). Ultrasonographic assessment of renal microcirculation is a new vision for the treatment of intensive care unit associated acute kidney injury. Eur. J. Med. Res..

[15] Sellitto S., Tarantino L., Barone F., Barone N., Perna A., Lucariello A., Guerra G., De Luca A., Filippelli A., Sellitto C. (2024). Contrast-Enhanced Ultrasound as a Diagnostic Procedure in Renal Diseases: A Case Report. G. Ital. Nefrol..

[16] Tsili A.C., Andriotis E., Gkeli M.G., Krokidis M., Stasinopoulou M., Varkarakis I.M., Moulopoulos L.A. (2021). Oncologic Imaging Subcommittee Working Group of the Hellenic Radiological Society. The role of imaging in the management of renal masses. Eur. J. Radiol..

[17] Zhu J., Li N., Zhao P., Wang Y., Song Q., Song L., Li Q., Luo Y. (2023). Contrast-enhanced ultrasound (CEUS) of benign and malignant renal tumors: Distinguishing CEUS features differ with tumor size. Cancer Med..

[18] Sun D., Wei C., Li Y., Lu Q., Zhang W., Hu B. (2016). Contrast-Enhanced Ultrasonography with Quantitative Analysis allows Differentiation of Renal Tumor Histotypes. Sci. Rep..

[19] Dipinto P., Canale V., Minelli R., Capuano M.A., Catalano O., Di Pierro G.B., Anceschi U., Perdonà S., Tufano A. (2024). Qualitative and quantitative characteristics of CEUS for renal cell carcinoma and angiomyolipoma: A narrative review. J. Ultrasound.

[20] Drelich K., Zbroja M., Cyranka W., Pustelniak O., Kopyto E., Kuczyńska M. (2021). The definitive role of CEUS in an ambiguous case of renal cell carcinoma. J. Ultrason..

[21] Stojanović N., Ignjatovic I., Kostov M., Mijović Z., Zivković S., Kosević B. (2013). Giant renal oncocytoma. Vojnosanit. Pregl..

[22] Tao L., Fan J., Zhan W., Li W., Lu J., Yang N., Ma B., Zhou W. (2022). Contrast-enhanced ultrasound manifestations of renal masses undetectable on conventional ultrasound. Front. Oncol..

[23] Atri M., Jang H.J., Kim T.K., Khalili K. (2022). Contrast-enhanced US of the Liver and Kidney: A Problem-solving Modality. Radiology.

[24] Tufano A., Drudi F.M., Angelini F., Polito E., Martino M., Granata A., Di Pierro G.B., Kutrolli E., Sampalmieri M., Canale V. (2022). Contrast-Enhanced Ultrasound (CEUS) in the Evaluation of Renal Masses with Histopathological Validation-Results from a Prospective Single-Center Study. Diagnostics.

[25] Lu Q., Xue L.Y., Huang B.J., Wang W.P., Li C.X. (2015). Histotype differentiation of hypo-echoic renal tumors on CEUS: Usefulness of enhancement homogeneity and intensity. Abdom. Imaging.

[26] Gerst S., Hann L.E., Li D., Gonen M., Tickoo S., Sohn M.J., Russo P. (2011). Evaluation of renal masses with contrast-enhanced ultrasound: Initial experience. AJR Am. J. Roentgenol..

[27] King K.G., Gulati M., Malhi H., Hwang D., Gill I.S., Cheng P.M., Grant E.G., Duddalwar V.A. (2015). Quantitative assessment of solid renal masses by contrast-enhanced ultrasound with time-intensity curves: How we do it. Abdom. Imaging.

[28] Wu Y., Du L., Li F., Zhang H., Cai Y., Jia X. (2013). Renal oncocytoma: Contrast-enhanced sonographic features. J. Ultrasound Med..

[29] Liu H., Cao H., Chen L., Fang L., Liu Y., Zhan J., Diao X., Chen Y. (2022). The quantitative evaluation of contrast-enhanced ultrasound in the differentiation of small renal cell carcinoma subtypes and angiomyolipoma. Quant. Imaging Med. Surg..

[30] Najafi A., Wildt M., Hainc N., Hohmann J. (2021). Evaluation of Cystic and Solid Renal Lesions with Contrast-Enhanced Ultrasound: A Retrospective Study. Ultrasound Int. Open.

[31] Nicolau C., Antunes N., Paño B., Sebastia C. (2021). Imaging Characterization of Renal Masses. Medicina.

[32] Kaur R., Juneja M., Mandal A.K. (2020). An overview of non-invasive imaging modalities for diagnosis of solid and cystic renal lesions. Med. Biol. Eng. Comput..

[33] Alrumayyan M., Raveendran L., Lawson K.A., Finelli A. (2023). Cystic Renal Masses: Old and New Paradigms. Urol. Clin. N. Am..

[34] Park B.K., Kim B., Kim S.H., Ko K., Lee H.M., Choi H.Y. (2007). Assessment of cystic renal masses based on Bosniak classification: Comparison of CT and contrast-enhanced US. Eur. J. Radiol..

[35] Cantisani V., Bertolotto M., Clevert D.A., Correas J.M., Drudi F.M., Fischer T., Gilja O.H., Granata A., Graumann O., Harvey C.J. (2021). EFSUMB 2020 Proposal for a Contrast-Enhanced Ultrasound-Adapted Bosniak Cyst Categorization–Position Statement. Ultraschall Med..

[36] Lan D., Qu H.-C., Li N., Zhu X.-W., Liu Y.-L., Liu C.-L. (2016). The Value of Contrast-Enhanced Ultrasonography and Contrast-Enhanced CT in the Diagnosis of Malignant Renal Cystic Lesions: A Meta-Analysis. PLoS ONE.

[37] Elbanna K.Y., Jang H.J., Kim T.K., Khalili K., Guimarães L.S., Atri M. (2021). The added value of contrast-enhanced ultrasound in evaluation of indeterminate small solid renal masses and risk stratification of cystic renal lesions. Eur. Radiol..

[38] Herms E., Weirich G., Maurer T., Wagenpfeil S., Preuss S., Sauter A., Heck M., Gärtner A., Hauner K., Autenrieth M. (2023). Ultrasound-based “CEUS-Bosniak” classification for cystic renal lesions: An 8-year clinical experience. World J. Urol..

[39] Möller K., Jenssen C., Correas J.M., Safai Zadeh E., Bertolotto M., Ignee A., Dong Y., Cantisani V., Dietrich C.F. (2023). CEUS Bosniak Classification—Time for Differentiation and Change in Renal Cyst Surveillance. Cancers.

[40] Sidhu P.S., Cantisani V., Dietrich C.F., Gilja O.H., Saftoiu A., Bartels E., Bertolotto M., Calliada F., Clevert D.A., Cosgrove D. (2018). The EFSUMB Guidelines and Recommendations for the Clinical Practice of Contrast-Enhanced Ultrasound (CEUS) in Non-Hepatic Applications: Update 2017 (Long Version). Ultraschall Med..

[41] Mazziotti S., Zimbaro F., Pandolfo A., Racchiusa S., Settineri N., Ascenti G. (2010). Usefulness of contrast-enhanced ultrasonography in the diagnosis of renal pseudotumors. Abdom. Imaging.

[42] Spiesecke P., Fischer T., Maxeiner A., Hamm B., Lerchbaumer M.H. (2021). Contrast-enhanced ultrasound (CEUS) reliably rules out neoplasm in developmental renal pseudotumor. Acta Radiol..

[43] Bertolotto M., Cicero C., Catalano O., Currò F., Derchi L.E. (2018). Solid Renal Tumors Isoenhancing to Kidneys on Contrast-Enhanced Sonography: Differentiation from Pseudomasses. J. Ultrasound Med..

[44] Fontanilla T., Minaya J., Cortés C., Hernando C.G., Arangüena R.P., Arriaga J., Carmona M.S., Alcolado A. (2012). Acute complicated pyelonephritis: Contrast-enhanced ultrasound. Abdom. Imaging.

[45] Marschner C.A., Ruebenthaler J., Schwarze V., Negrão de Figueiredo G., Zhang L., Clevert D.A. (2020). Comparison of computed tomography (CT), magnetic resonance imaging (MRI) and contrast-enhanced ultrasound (CEUS) in the evaluation of unclear renal lesions. RöFo.

[46] Dietrich C.F., Nolsøe C.P., Barr R.G., Berzigotti A., Burns P.N., Cantisani V., Chammas M.C., Chaubal N., Choi B.I., Clevert D.A. (2020). Guidelines and Good Clinical Practice Recommendations for Contrast-Enhanced Ultrasound (CEUS) in the Liver-Update 2020 WFUMB in Cooperation with EFSUMB, AFSUMB, AIUM, and FLAUS. Ultrasound Med. Biol..

[47] El-Bandar N., Lerchbaumer M.H., Peters R., Maxeiner A., Kotsch K., Sattler A., Miller K., Schlomm T., Hamm B., Budde K. (2022). Kidney Perfusion in Contrast-Enhanced Ultrasound (CEUS) Correlates with Renal Function in Living Kidney Donors. J. Clin. Med..

[48] Back S.J., Acharya P.T., Bellah R.D., Cohen H.L., Darge K., Deganello A., Harkanyi Z., Ključevšek D., Ntoulia A., Paltiel H.J. (2021). Contrast-enhanced ultrasound of the kidneys and adrenals in children. Pediatr. Radiol..

[49] Kapur J., Oscar H. (2015). Contrast-enhanced ultrasound of kidneys in children with renal failure. J. Med. Ultrasound.

[50] Stemkens B., Prins F.M., Bruijnen T., Kerkmeijer L.G.W., Lagendijk J.J.W., van den Berg C.A.T., Tijssen R.H.N. (2019). A dual-purpose MRI acquisition to combine 4D-MRI and dynamic contrast-enhanced imaging for abdominal radiotherapy planning. Phys. Med. Biol..

[51] Reiner C.S., Goetti R., Eberli D., Klotz E., Boss A., Pfammatter T., Frauenfelder T., Moch H., Sulser T., Alkadhi H. (2012). CT perfusion of renal cell carcinoma: Impact of volume coverage on quantitative analysis. Investig. Radiol..

[52] Zhang D., Wang Y., Yang F., Mao Y., Mu J., Zhao L., Xu W. (2023). Diagnostic Value of Multi-Mode Ultrasonic Flow Imaging Examination in Solid Renal Tumors of Different Sizes. J. Clin. Med..

[53] Bertolotto M., Bucci S., Valentino M., Currò F., Sachs C., Cova M.A. (2018). Contrast-enhanced ultrasound for characterizing renal masses. Eur. J. Radiol..

[54] Mao Y., Mu J., Zhao J., Yang F., Zhao L. (2022). The comparative study of color doppler flow imaging, superb microvascular imaging, contrast-enhanced ultrasound micro flow imaging in blood flow analysis of solid renal mass. Cancer Imaging.

[55] Kuang X., Wang H., Chai W., Yuan H., He T., Shi M., Jiang T. (2024). Clinical diagnostic value of contrast-enhanced ultrasound combined with microflow imaging in benign and malignant renal tumors: A retrospective cohort study. Biomol. Biomed..

[56] Müller-Peltzer K., Rübenthaler J., Negrao de Figueiredo G., Clevert D.A. (2018). CEUS–Diagnostik benigner Leberläsionen [CEUS–diagnosis of benign liver lesions]. Radiologe.

[57] Chiorean L., Tana C., Braden B., Caraiani C., Sparchez Z., Cui X.W., Baum U., Dietrich C.F. (2016). Advantages and Limitations of Focal Liver Lesion Assessment with Ultrasound Contrast Agents: Comments on the European Federation of Societies for Ultrasound in Medicine and Biology (EFSUMB) Guidelines. Med. Princ. Pract..

[58] Inzerillo A., Meloni M.F., Taibbi A., Bartolotta T.V. (2022). Loco-regional treatment of hepatocellular carcinoma: Role of contrast-enhanced ultrasonography. World J. Hepatol..

[59] Guo F., Hu B., Chen L., Li J. (2019). Clinical application of contrast-enhanced ultrasound after percutaneous renal tumor ablation. Br. J. Radiol..

[60] Vovdenko S., Ali S., Ali H., Taratkin M., Morozov A., Suvorov A., Khabib D., Rapoport L., Bezrukov E. (2024). Contrast-enhanced ultrasound (CEUS) as a follow-up method after the focal treatment of renal tumors: Systematic review and meta-analysis. Int. Urol. Nephrol..

